# Athletes and Coaches through the COVID-19 Pandemic: A Qualitative View of Goal Management

**DOI:** 10.3390/ijerph19095085

**Published:** 2022-04-21

**Authors:** Sergio Costa, Eugenio De Gregorio, Lisa Zurzolo, Giampaolo Santi, Edoardo Giorgio Ciofi, Francesco Di Gruttola, Luana Morgilli, Cristina Montesano, Francesca Cavallerio, Maurizio Bertollo, Selenia di Fronso

**Affiliations:** 1 Independent Researcher, 00165 Rome, Italy; costasergio@hotmail.it (S.C.); giampaolo.santi@libero.it (G.S.); e.ciofi@gmail.com (E.G.C.); luana.morgilli@gmail.com (L.M.); 2 Department of Life and Health Sciences, Link Campus University, 00165 Rome, Italy; e.degregorio@unilink.it; 3 Department of Education, University of Genoa, 16128 Genova, Italy; lisazurzo@hotmail.com; 4 IMT School for Advanced Studies Lucca, 55100 Lucca, Italy; francesco.digruttola@gmail.com; 5 Department of Neuroscience, Imaging and Clinical Sciences, University “G. d’Annunzio” of Chieti-Pescara, 66100 Chieti, Italy; cristina.montesano@unich.it; 6 School of Psychology and Sport Sciences, Anglia Ruskin University, Cambridge CB1 1PT, UK; francesca.cavallerio@aru.ac.uk; 7 Department of Medicine and Aging Sciences, University “G. d’Annunzio” of Chieti-Pescara, 66100 Chieti, Italy; s.difronso@unich.it

**Keywords:** goal setting, goal adjustment, lockdown, qualitative survey, reflexive thematic analysis

## Abstract

Since the end of 2019 and throughout 2020, the world has been devastated by the COVID-19 pandemic. The sports world suddenly had to deal with a massive reorganization of events with important implications for the physical and psychological preparation of athletes and coaches. The purpose of this study was to explore how these changes impacted coaches’ and athletes’ goal-setting strategies and their experience of goal adjustment. As part of a wider mixed-method project involving 2162 coaches and 1354 athletes, an online qualitative survey was used, and data collected were analyzed using reflexive thematic analysis. Findings highlighted three overarching themes, in response to goal adjustment: “Moving on toward new goals”, “Letting go of goals”, and “Trying to hold on”, with several themes and sub-themes identifying different nuances of athletes’ and coaches’ experiences. The implications of such findings for the mental preparation of high-level athletes are discussed in two ways. Firstly, in light of existing literature on goal setting from an applied perspective; secondly, in the broader perspective of the sports culture and the application of our themes to other challenging moments that sports professionals might encounter.

## 1. Introduction

The year of the Olympic Games is normally seen as the realization of the four-years-periodization of specific, measurable, achievable, realistic, timely, and self-determined (SMARTS) goals for both athletes and coaches [1]. Some of these athletes and coaches could have fulfilled their plans and dreams of competing in Tokyo 2020, and others could have reached their own annual goals of competing at the international and/or national level [2]. However, in the last months of 2019, and in the early ones of 2020, the SARS-CoV-2 virus affected the entire world [3], including sports, forcing people to find at-home alternatives to exercise [4,5]. Both professional and amateur athletes and coaches were constrained at home in isolation, without the possibility to train and compete in their usual environments, with potential impacts on their lives and career [6]. Thus, they had to change their lifestyle and routines, interpersonal relationships, financial status (e.g., loss of a job or of a sponsor), as well as reorganize their aspirations and goals [7]. Existing research showed that in some situations, it can be more productive to abandon an important goal and allocate resources to alternative goals [8], although athletes may struggle to do this in the middle of the competitive sports season.

A goal is defined as “an internal representation of desired result, event or process” [9] (p. 485). It may refer to a result that athletes or coaches want to obtain (e.g., winning a trophy or a gold medal), to the identification of a specific standard to be achieved (e.g., performing one’s own personal best in a marathon), or to the strategy needed to attain a performance goal, for example focusing on a tactical aspect of the game [10]. 

A prominent theory that has been extensively employed in goal-setting research and practice is the goal-setting theory (GST), which explains the relationship between conscious goals and task performance [11,12]. The literature of the past 30 years on goal setting in sports suggests that setting specific and challenging, long- and short-term goals is an effective way to enhance performance in sport [10,13]. Moreover, goal setting is one of the most important strategies to improve athletes’ motivation and identity [14]. Yet, often not all the types of goals (i.e., process, performance and outcome) are taken into consideration and assessed during a season by athletes and their coaches. For instance, Forsblom et al. [15] investigated the perceived goal-setting practices of athletes across a competitive season, showing that the achievement of the outcome goal was always evaluated, whereas the attainment of process and performance goals was evaluated only occasionally. However, in their study, the authors recognized some limitations, such as the small sample, the involvement of only female participants, and the need to also include their coaches and investigate different levels of expertise. 

A recent systematic review of goal-setting interventions in sports focused on athletes, found inconclusive evidence and limited support for several aspects of goal-setting theory in applied sports contexts [16]. The authors suggested involving a wider range of theories of goal setting to provide a more comprehensive understanding of this area. For instance, Jeong and colleagues indicated the self-concordance model [17] as an example to examine motivation underpinning goal pursuit, its impact on goal striving and attainment, as well as psychological well-being after goal attainment (or failure, or disengagement). This model has been shown to be relevant to understanding how coaches can support adaptive goal striving [18].

Another recent model, the tripartite model of goal striving, which integrates goal motivation and goal regulation, can be used to explain the mechanisms behind goal adjustment, re-engagement and disengagement [8]. The authors suggest that self-regulatory responses depend on two key mechanisms: the motives for goal pursuit, and a metacognitive strategy called mental contrasting with implementation intentions.

To our knowledge, the only two studies that analyzed athletes’ goals during COVID-19 were conducted by Mascret [19] and by Martinez-Gonzalez and colleagues [20] Specifically, Mascret used a quantitative method to investigate whether the confinement and the physical exercise restrictions, due to the COVID-19 pandemic situation, may have impacted the achievement of athletes’ goals. Findings reinforced the general assumption that “different achievement goals may be better suited for different types of situations” [21] (p. 708). Moreover, it was found that self-avoidance goals (where the athletes try to avoid self-based incompetence) are not always maladaptive, and that, in the situation of confinement, it might be beneficial to shift among multiple goals. Martinez-Gonzalex et al. examined the effect of the COVID-19 lockdown on changes in athletes’ reported subjective vitality and goal motives (autonomous and controlled) over time (i.e., pre-lockdown and during lockdown). Findings suggest a negative impact of the COVID-19 lockdown on athletes’ goal motives and subjective vitality. Results also support the hypothesized mediational role of autonomous goal motives in the relationship between resilience and subjective vitality during the lockdown. As such, findings confirm not only the relevance of resilience to a key feature of the athletes’ eudaimonic well-being but also the importance of enhancing their autonomous goal striving. While informative, these studies fail to answer Jeong et al.’s [16] call for future research to consider empirical approaches that are based on coaches’ experiential knowledge and the recommendation to include qualitative approaches to identify practitioners’, coaches’, and athletes’ perspectives on goal setting.

In a similar fashion, to date, research investigating the effects of the COVID-19 pandemic in sports mainly adopted a quantitative approach, with only a few exceptions [22,23], despite a growing interest in—and need for—qualitative studies in the field of sports psychology [24]. In line with Jeong et al.’s [16] suggestion for further qualitative studies on goal setting, the present study aimed to explore Italian coaches’ and athletes’ experiences of goal-setting adjustment during the first pandemic lockdown (Spring 2020) adopting a qualitative stance. From a qualitative perspective, with this study, we also aimed to add to the literature on how athletes and coaches have managed the cancellation of championships and the postponement of sports competitions, as well as the prolonged Italian lockdown. The research question guiding the study was, “how has the first emergency lockdown influenced athletes and coaches’ experiences of goal setting adjustment?”

As suggested by previous research [2,25], the perceived significance of a change event is related to athletic identity [26,27] as well as to the impact and imbalance it creates in the athlete’s career. By conceptualizing this pandemic situation as an injury recovery phase, rather than a break without the possibility to train and compete [6], we expected that it could also impact the definition of new goals, especially for those who thought to return to sports competition soon.

## 2. Materials and Methods

### 2.1. Philosophical Assumptions

This study—part of a wider mixed-method project—is underpinned by an interpretivist paradigm, with a relativist ontology and a constructivist epistemology [28]. This means that in this work we consider the nature of reality to be multiple, with subjective realities being constructed in each individual mind, and therefore knowledge is co-constructed, with the researchers’ own experience mediating what can be understood [29]. In line with these philosophical assumptions, we understood the data we collected as an opportunity to explore participants’ points of view, experience and subjectivity, with an awareness that our analysis would be the result of possible (re)interpretations and positioning of the researchers involved [30,31].

### 2.2. Participants

Following ethical approval obtained from the ethics committee for biomedical research of Chieti-Pescara University (ID richiam7px), in line with the ethical principles of the Declaration of Helsinki [32], recruitment took place using both social media platforms (e.g., Facebook, Instagram) and gatekeepers (e.g., national governing bodies, sports clubs). Following Patton [33], a combined purposeful sampling strategy, beginning with maximum variation (i.e., choosing a range of cases to obtain variation on dimensions under study), and followed up by chain sampling (i.e., participants shared the link of the survey to relevant contacts) was used. Expertise categorization was based on Swann et al.’s [34] classification and participants were asked to self-report if they were competing/coaching at a local, regional, national or international level. Therefore, scores for the local level were assigned to the low-experts category and scores for regional, national and international levels were assigned to the high-experts category. Overall, 3516 participants were surveyed. Specifically, 2162 participants (women = 713; mean age = 41 ± 18.32 ys), working within individual (e.g., skating, swimming, gymnastics and others; n = 865, 40%) and team (e.g., soccer, volley, rugby and others; n = 1297, 60%) sports, with different reported levels of expertise (1324 low expert, 838 high expert) were recruited among Italian coaches. As for athletes, 1354 participants (women = 712; mean age = 27.50 ± 12.76 ys), competing in individual (e.g., swimming, tennis, athletics and others; n = 708, 52%) or team (e.g., volley, soccer, basketball and others; n = 646, 48%) sports, with 777 athletes reporting to be low experts, and 577 defining themselves as high experts. However, participants who eloquently answered our questions were 1587 coaches out of 2162 and 1808 athletes out of 1354 (see also the data analysis).

### 2.3. Procedure and Materials

The study was conducted between 22 March 2020—three weeks after the start of the forced confinement caused by the COVID-19 health emergency in Italy—and 4 May 2020, when the Italian government partially eased the lockdown regimen. Considering the study’s philosophical underpinnings, an online qualitative survey [35] was the chosen method. Qualitative surveys have been described as a data collection tool, which uses a series of pre-determined and fixed open-ended questions that participants need to answer in writing [36]. One recognized benefit of this method of data collection is the possibility to combine the openness of the questions—and therefore a variety of answers—with standardization due to the fixed set of questions posed. They often allow participants to proceed at their own pace and typically they also provide anonymity [37]. Qualitative surveys can be conducted online, which was a fundamental characteristic required to conduct research during the pandemic.

Participants received a link to the online survey and, following a brief description of the study and the provision of informed consent, they were asked to complete the online survey. This was divided into three sections: (a) sociodemographic information (e.g., gender, nationality, date of birth) (b) information on the sport practiced (e.g., type of sport, level of activity), and (c) information on the use of goal setting before and after the COVID-19 lockdown. In particular, in the third section of the survey, two open-ended questions were asked to both athletes and coaches. These questions were: (a) “*Before COVID-19 lockdown, did you set your seasonal goals? If so, which ones*?”, and (b) “*Do you think you have to change your goals due to COVID-19 lockdown? If so, how has the lockdown influenced your experiences of goal setting adjustment?*”.

### 2.4. Data Analysis

We conducted a reflexive thematic analysis [38] to examine the data collected, identifying patterns in relation to the experience of goal adjustment for athletes and coaches in response to the first COVID-19 lockdown in Italy. In line with Braun and Clarke [38], we intended for this approach to be “creative, reflexive, and subjective, with researcher subjectivity understood as a resource rather than a potential threat to knowledge production” (p. 591). In this sense, the qualitative analysis aims to reconstruct the processes of meaning-making. The data are positioned in a specific context, located in the story and in the stories of those who tell the experiences. 

Our analysis started with the initial research question, as its formulation highlighted the aim to explore the experience of the participants and the criteria for sense-making, perceptions, motivations and points of view [39]. As part of a pre-analytic moment, we adopted what Braun and Clarke [36] call a selective coding approach, in order to reduce our dataset and identify the analytic concepts we were looking for. Given our large sample size combined with the method of data collection, we had participants who eloquently answered our question, while others simply answered with a “No”. Therefore, when focusing on the experience of goal adjustment post-lockdown, we only selected the answers that gave us more information, excluding 453 monosyllabic answers “No” by athletes and 575 by coaches. Still, it is important to highlight that a third of our sample simply answered that lockdown was not going to impact their goals. 

Once the dataset had been selected, FC and EDG started a process of prolonged data immersion, while familiarizing themselves with the data. Moving onto the coding phase, the ideas expressed that related to the research question were ‘labelled’ through the whole dataset (athletes and coaches). The resulting codes were then examined and collated in order to identify shared patterns of meaning (i.e., underpinned by a ‘core concept’, [38], (p. 593). While generating these potential themes, attention was paid to those that only related to codes from the athletes’ or the coaches’ responses. While these were not many, we considered it could be an important aspect from an applied perspective to ensure we highlighted any specific theme. Once candidate themes had been generated, they were shared with the wider team that acted as critical friends. The group discussion allowed the analysis to start being shaped not only in a data-driven way but also—thanks to the expertise of the team members—candidate overarching themes were identified using a theory-driven approach [36]. Following these discussions, themes were reviewed and adjusted, clarifying their hierarchical relationship, and then definitions and names were created for each theme, paying attention to highlight the focus of each theme and the ‘story’ of each one, as well as how they related to each other in answering the research question. The results of this analysis are presented in the following section, together with a thematic map that aims to summarize and clarify relationships between overarching themes, themes, and generated sub-themes.

### 2.5. Quality Criteria

To ensure rigor in the conduction of this study, from data collection through to the representation of results, we adopted a non-foundational approach [40], which lets go of the idea of ‘universal criteria’ to judge the quality of qualitative research and instead, focuses on a study-specific list of ‘characterizing traits’ to ensure rigor and quality [41]. In line with this approach, we selected five quality criteria from the list suggested by Smith and Caddick [42]: substantive contribution, impact, width, coherence and transparency. ‘Substantive contribution’ refers to the way we aimed, for this study, to be useful in our understanding of the experience of lockdown for athletes and coaches, helping to see possible consequences and/or prevent further negative experiences in the future, and how—in order to achieve this—we embedded our interpretation in existing research and theories. With ‘impact’, we aimed for the study to affect the work of sports professionals, encouraging them to act in proactive and constructive ways to prevent—where possible—negative situations. We also wanted to encourage researchers to look ‘across’ different areas of research to transfer knowledge in order to support athletes in challenging situations. The criteria of ‘width’ relates to the comprehensiveness of the data we collected. While recognizing the possible limitations of the chosen method of data collection, we are also aware of the size of our sample, which allowed for richness in our dataset, and we also strived to provide readers with thoughtful interpretations of the evidence. With regard to ‘coherence’, our work aimed to present a meaningful picture of the phenomenon we studied, both in relation to how our themes related to the research question and to how our interpretations integrate and add to existing research. Finally, we worked in order to make our work ‘transparent’ through the use of several critical friends, who acted as a sounding board and ensured a depth of analysis, and also reflection and exploration of alternative interpretations of our data. With these points in mind, we invite the reader to explore our results in response to the research question, “How has the first emergency lockdown influenced athletes and coaches’ experiences of goal setting adjustment?”

## 3. Results

**Figure 1:**
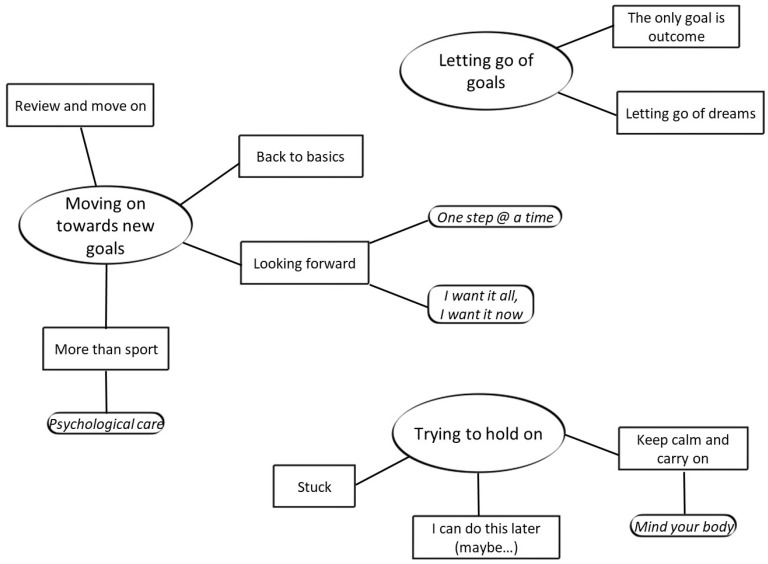
A thematic map representing the three overarching themes (oval shapes), the themes (rectangles), and the sub-themes (smaller shapes) resulting from our analysis and their relationship.

Following reflexive thematic analysis, we identified nine themes, organized underneath three overarching themes. There are also four sub-themes that will be illustrated below. Figure 1 provides a thematic map summarizing the results in response to the research question, “*How has the first emergency lockdown influenced athletes and coaches’ experiences of goal setting adjustment*?” The three overarching themes—common to both athletes and coaches—identified are: “Moving on toward new goals”, “Letting go of goals”, and “Trying to hold on”.

### 3.1. Moving on towards New Goals

This overarching theme focuses on those experiences of athletes and coaches adapting their work, despite the challenging and unprecedented situation they lived in. Participants identified alternative goals on which they were able to focus, thus highlighting an ability for continuous growth.

#### 3.1.1. Review and Move On

For many participants, the lockdown was simply considered an obstacle (like an injury), which required them to review and adjust their plans and future goals, without attaching any specific meaning/reaction to it. One athlete simply said that their plan was to “change competitions—as the ones I wanted to take part in were cancelled—and modify my conditioning”, while another mentioned the need to adapt to new post-pandemic requirements: “I’ll have to resize [my goals] based on what will actually be allowed following this emergency”. 

For others, the impact—and consequent need to adjust—went beyond just reviews of calendars. For example, one athlete said: “For now, my goals are to stay healthy—as I can’t go to the swimming pool or to the physio like I was used to—and to stay fit. As per competitions, we’ll come up with a new plan as soon as new dates will become official, both for international meetings, Olympics, and Euros”.

Coaches had similar thoughts, knowing they would have to review goals based on their athletes’ situations. For example, one coach said, “Yes, I will have to review everything based on how gymnasts’ condition will be once back and on how the National Governing Bodies will reprogram competitions”. 

For lower-level coaches, financial aspects might also have been at stake, like for this coach, who explained: “Surely there will be an important decrease in athletes, and so for the moment I am just waiting for the green light to start again, and then once I see how many girls I’ll have back, I’ll plan what to do, but I am pretty sure I will organize a summer camp”.

#### 3.1.2. Back to Basics

Some of our athletes and coaches were able to appreciate the possibility of focusing on process goals, going back to the basics of training, improving technique rather than a game, or simply going back to practicing sport just for the pleasure of it, rather than trying to achieve something. One coach said, “We will organize training differently, moving the focus away from performance”. Similarly, team sports coaches had to adapt to the isolation, as one coach explained: “While we cannot work on the group, we are training individual strength and skills”. This point was echoed by athletes, with one explaining, “I am focusing more on individual training, as I cannot count on the team now”. One individual athlete explained their adapted approach: “I am maintaining the same movements as in the practice, training in front of the mirror, visualizing different situations and doing preparatory exercises”.

This theme represents a more positive appraisal of lockdown, which is not seen as “having to be negative”, but almost as a respite from the non-stop performance culture of sports. As some athletes explained, “I will train for fun and not with a competition in mind”, and “I postponed everything. I am training anyway simply because I enjoy it”.

#### 3.1.3. Looking Forward

This theme refers to those athletes who adapted their goals by focusing on the following season. With the focus of starting again, looking at the future appears logical considering the circumstances, it was quite clearly represented by two contrasting approaches, highlighted in the sub-themes below.

##### One Step @ a Time

This sub-theme identifies the idea that by focusing on the following season, athletes/coaches had to take care of themselves/their athletes when engaging with training again, increasing loads gradually, and even accepting to decrease the level of their future goals. As one coach explained, “we will go back to a long-term planning”. While another highlighted the adjustments needed: “We will have to let go of squad training sessions, as Nordic competitions are unlikely to be organized. As soon as it will be possible to start again, I will have to start with training sessions for members but with a slower and balanced intensity”.

These words were echoed by athletes, for example, one said that it was important to “understand that once we will be able to go back [to training] lots of calm and patience will be needed, both with regards to my body and to my performance”.

##### I Want It All, I Want It Now

On the other hand, several athletes (and some coaches) showed attitudes that fell completely in line with the performance narrative and dominant values of “more is better”, seeing an immediate increase in training and intensity as fundamental to come back from lockdown. The emergency and the break that came with it are almost not recognized, in a view that ignores anything that is not “the best” and “winning”.

Using metaphors related to war scenarios, one athlete said his goal was to “be ready and with a knife between my teeth once everything will be over”, while others mentioned having to “make up for lost time” and to “speed up improvement once I’ll start competing again”. These ideas were also echoed by a few coaches—in stark contrast with the views represented in the previous sub-theme—stating that, “We will have to speed up our rhythm as soon as we’ll finally be allowed to be back on the pitch”, or simply saying that once back, they’ll have to “pick things up where they left”, failing to acknowledge any consequence—physical and/or psychological—of the pandemic.

#### 3.1.4. More Than Sport

While the majority of participants’ focus was on training, when asked how their goals would be adjusted after the lockdown, some athletes and coaches recognized that at times sport is not the most important thing in life and that other aspects need to become a priority. This shift in focus and re-engagement with goals in different areas can be heard in the words of this athlete, who simply said that “Nowadays there are things that are more important than sport”, or “I accept this situation. Health is more important”. Similarly, coaches also saw this suspension in time as an opportunity for personal development, as one coach explained: “I will complement my [now part-time] job with new personal goals, like a master’s degree”. Keeping a focus on their athletes’ goals instead, one coach highlighted all the aspects impacting new planning: “Before I can decide on new goals, I will have to evaluate—once the emergency is over—in which condition my group will be, physically, mentally, but also logistically”.

##### Psychological Care

More specifically, many coaches’ focus was on the effects of the stop on the mindset of their athletes. The importance of working on mindset, increasing motivation, and focusing on team building is brought to the forefront, not just from a performance perspective, but from a more holistic one, highlighting qualities of care in the coach-athlete relationship. Several coaches’ responses focused on this aspect, explaining that they were “keeping the team together from a distance and making them feel I am there, and I care”, and how “now the focus is ensuring their [young athletes’] wellbeing through sport. Everything else can wait”, and “Now we need to stay in contact with the athletes and not leave them alone”. Another coach highlighted their plan once going back: “[I will have to] reorganize the activities for when we’ll be back, focusing on rebuilding the team and get athletes back to being used to train like they did before lockdown. Motivation and engagement will be fundamental. Personally speaking, I’d completely let go of the idea of start competing as soon as possible, I don’t think it is necessary”.

### 3.2. Letting Go of Goals

This overarching theme presents the experiences of those athletes and coaches that realized the unattainable nature of their current goals and were able to disengage from them, avoiding negative consequences, such as failure and low self-esteem. Yet, the disengagement appeared to happen in two different ways, which is highlighted in the following themes.

#### 3.2.1. Letting Go of Dreams

For some participants, adapting goals meant having to let go of plans and dreams they had; while a veil of sadness was apparent in the idea of letting go, on the other hand, this seemed to leave space for future goals. For example, one athlete explained that “For now, it’s a one-year break and I am just training [with no specific goal]” and another one said, “Yes, my goals are cancelled at the moment, I am focusing on health and fitness”. Coaches also recognized the need to restart, as one explained that “The NGB decided this for us, we are starting from zero again”. In some situations, this also meant letting go of sport completely, considering retirement due to the forced stop and its impact on their age/career. For example, one athlete said, “I think next year it will be my last one as a player, mainly to help “bridging” the young ones into the older group, and just focus my activity on coaching youngsters”.

#### 3.2.2. The Only Goal Is Outcome

For many participants, though, goals had to be changed because competitions had been cancelled and they identified goals with “competition”. This theme highlights the focus on performance outcomes and a lack of understanding of different types of goals. This athlete’s perspective summarizes many participants’ expressed sense of frustration: “There are no competitions, so this year is wasted”. While the negative effect a lack of goals might have on athletes’ motivation is imaginable, it seemed even worse when the same concepts were echoed in the words of the coaches, like the one who said, “Competitions have been cancelled, so no goals anymore”, or the coach who explained: “We have to wait and see how the NGB will review the competition calendar. It might even be that these Championships are cancelled, for now they are suspended. If they confirm them, we’ll try and prepare at our best”.

### 3.3. Trying to Hold On

This overarching theme highlights the experience of those participants who struggled to let go of their goal and instead somehow persisted with it. The idea of “giving up” has often been depicted as negative in sport, with an unspoken concept of weakness attached to it. While holding on to previous goals does not need to be considered a priori as negative, the risk of frustration, and a loss of self-esteem and control in the face of unattainable goals that cannot be let go is present. The themes identified in our results highlight different experiences of participants who were not able or did not want to let go of their goals and the following effect.

#### 3.3.1. Keep Calm and Carry On

Some of the athletes and coaches managed to stay in the present, simply taking the experience day by day, trying not to worry for the future, in an approach that appears grounded, and even if at times a little naïve in the lack of adjustment of their goals. While one coach mentioned, “We have to start from where we left so that we can still reach our original goals”, others simply counted on the summer ahead, mentioning they would have to “work a few more weeks at the end of the season” or “extend training until the end of July”. One coach just said they aimed at “staying calm and waiting for everything to be over”. In a similar vein, one athlete, when asked if they were planning to change their goals, said, “It depends, we’ll see once this situation is over how the year will continue. For now, I just keep training and I don’t worry about it”, while another one explained: “It depends, for the moment we just have to adapt to this situation in order to improve our fitness (the only training doable at home) and understand when we will be able to go back to normality. If we won’t start playing again by mid-May or those competitions currently suspended won’t take place, then I’ll have to change my goals”.

##### Mind Your Body

This athlete-specific sub-theme highlights a focus on staying in the present, which appears to be related to nutrition, and how athletes know they have to pay attention to it, considering their overall physical activity decreased. One athlete mentioned, “I try to control more what I eat and train at my best at home”, while others mentioned the need to “modify [my] eating plan” and try to “stay as fit as possible with regards to weight”.

#### 3.3.2. I Can Do This Later (Maybe…)

Many athletes and coaches commented on the fact that their goals only needed to be postponed. For example, one coach said, “Goals will simply be postponed with an exam to next season”, and another simply mentioned that they would “Keep the group training and just move the goals to next year”. Likewise, one athlete explained, “My goals stay the same for now, and then I’ll aim to get better and better. I simply postponed the time when I will reach these and future goals”.

Yet, this often meant an impact on the psychological state of athletes, who worried about a lack of motivation, the feeling of fatigue, thinking about having to get fit again or even being afraid not to “make it” past lockdown. One athlete expressed their worry, saying, “Today I had to let go a little, I am afraid I won’t reach it [the goal] any longer, I am feeling very negative and lonely in sticking to my goals”. Another athlete explained how, “goals stay the same but with parks being closed training for endurance running has become impossible unless you have a treadmill at home! I hope the level of conditioning I had achieved before won’t worsen too much”.

#### 3.3.3. Stuck

Many participants described a very negative experience of lockdown, due to an overall feeling of uncertainty, lack of control, and the impossibility to decide for themselves—as the situation was bigger than them. The uncertainty, in turn, created negative feelings of loneliness, missing teammates, frustration, a sense of being lost, and lack of control. For example, one athlete explained, “I cannot know when this will be over, I cannot know which other goals might be reachable and which will be the criteria to access these goals”. This athlete also highlights the misconception of seeing competitions as the only goal. Another athlete, still holding on to his goal, shows a sense of uncertainty saying, “The goal of winning one competition remains, but there is less time to get into the top 15 athletes, and so I am not sure I’ll be able to achieve my goal”. The sense of depending on others’ decisions was impending on many. For example, one athlete said, “My goal is linked to external factors on which we have no control at all, and so everything depends on that”.

Similar feelings of uncertainty and lack of control were presented by coaches, with one saying, “We are waiting for the NGB to decide”, while another one lamented that “The championships starting again or not is not on me’ and one also explained that ‘I don’t even know if we’ll be allowed to conclude our season, so as of today it is impossible for me to even think about how I could modify the goals”.

## 4. Discussion

The aim of the present study was to investigate how the first emergency lockdown influenced Italian athletes’ and coaches’ experiences of goal-setting adjustment. Specifically, we examined whether and how they changed goals due to the closure and postponement of sports competitions, and the prolonged, ‘draconian’, Italian lockdown. While it is renowned that effective goal setting can help athletes and coaches in a healthy pursuit of excellence [15], the data collected in this study highlight the challenges encountered in determining foreseeable goals due to the current uncertainties and confusion associated with the pandemic [22]. While this situation was certainly true at the time of data collection (i.e., spring 2020), we are aware that this feeling of uncertainty is likely to be extended for a long time [43], therefore results from this study are not just useful to understand ‘what happened’ but have the potential for impact on athletes’ and coaches’ lives nowadays. 

Our findings highlighted three overarching themes, in response to goal adjustment: “Moving on toward new goals”, “Letting go of goals”, and “Trying to hold on”. These themes align with existing literature on goal setting, and specifically with Ntoumanis and Sedikides’ [8] tripartite model of goal striving. In this model, the authors explain how the constructs of goal re-engagement, goal disengagement and goal persistence are influenced and affect emotional, cognitive, and performance consequences. These processes are reflected in the themes identified in this study. For example, when “Moving on toward new goals”, participants highlighted an ability to self-regulate, identify alternative goals, and consecutively engage in actions to achieve the new/revised goals. The theme “Back to basics” specifically highlights how participants’ solutions to move on were to refocus on process goals. Indeed, from an applied sports psychology perspective, process goals help athletes to maintain attentional focus; however, some athletes may need to develop process goals around maintaining motivation when it comes to certain exercises, and it can also increase effort and enjoyment [44].

According to Healy and colleagues [45], the ability to evaluate and change goals is linked to the ability to overcome a failure or a difficult period, such as the one that athletes and coaches experienced during the COVID-19 lockdown. To avoid negative feelings, follow-up and goal evaluation should be done throughout the season, and not only at the end of it. In this way, the progress toward each goal can be consistently monitored, and the action plan adjusted when needed [11,12,15,46], even in situations of forced stop, such as in the current pandemic.

Athletes’ and coaches’ adoption of a “Letting go of goals’’ approach resonates with the idea of goal disengagement, which Wrosch and colleagues [47] defined as a self-regulatory process through which a person withdraws their efforts from a goal that is recognized as out of reach. Several researchers reported beneficial effects of goal disengagement (e.g., avoiding psychological distress of failure, improving subjective wellbeing; [48,49]). Nonetheless, while goal disengagement is not a negative approach in itself, our results highlighted how—for many participants—goals became unattainable as a consequence of an imperfect goal-setting strategy before the health emergency, with a predominance of outcome goals being set. The literature on goal setting in sports has instead highlighted the combination of process, performance and outcome goals as the best strategy [50]. For example, Burton [51] found that setting a performance goal in combination with an outcome goal resulted in superior performance than setting an outcome goal alone. If an outcome goal requires several months to complete, it would be useful to define what should be achieved halfway through the process. The decision to let go or postpone goals may have had a significant impact on athletes or coaches, for example, some of them could have lost athletic identity, motivation, and meaning [52], considering a premature retirement to escape the overwhelming physical and mental requirements associated with the undue year of preparation [22]. Moreover, Ruffault et al. [53], during the COVID-19 lockdown, highlighted lower motivation scores, for example, for athletes without a specific training program. If we consider these findings in comparison with our results and in the framework of the goal-setting theory, we shall remember that the specificity and precision, with which we define a goal, are crucial for the quality of the performance, as well as for the allocation of attention and the necessary motivation [45,54]. Moreover, in their recent review, Jeong et al. [16] reminded us that Locke and Latham [12] suggested that goal specificity should be combined with goal difficulty for effective goal setting.

Our third overarching theme, “Trying to hold on”, reflects Ntoumanis and Sedikides’ [8] concept of ‘goal persistence’. These authors explain how this regulatory response—which influences goal attainment, as well as effective, cognitive, and performance consequences—is affected by individual differences and traits. This point is clearly mirrored in the themes identified in our analysis. On one hand, the theme “Keep calm and carry on” represents those participants who were able to set up an appropriate goal-setting strategy before the emergency, and therefore during the lockdown were able to stay focused on their previously set goals, without having to face the need to review them yet. On the other hand, “Stuck” and “I can do this later (maybe…)” portray a less positive experience, characterized by uncertainty, confusion, and stress.

The feelings of uncertainty described above may lead to increased psychological distress in both athletes and coaches [55,56]. For example, Pillay et al. [57] revealed that in this period, many athletes reported feeling depressed (52%) and have struggled to keep themselves motivated to exercise (55%). Ruffault and colleagues [53], partially confirming the results from di Fronso and colleagues [55], have registered higher levels of anxiety for females, younger athletes, athletes practicing and competing at the highest level, and athletes without a training program during the lockdown. To cope with these feelings and maintain a high level of well-being, some psychological interventions have been implemented around the world during the pandemics, such as webinars and training online, see for instance [58,59,60].

Moving our interpretation of results beyond goal-setting theories and framework, two themes and their sub-themes appeared to be of interest when trying to contextualize this study in the broader sports context. Among those participants whose approach fell under the “moving on” process, many mentioned the idea of focusing on the following season, the return to sport as we knew it. Yet, two very distinct approaches to this new season were identified, “I want it all, I want it now” and “One step @ a time”. The former theme could be seen as expressing values that studies in the narrative inquiry tradition recognize as reflecting what is called a ‘performance narrative’ [61]. This narrative reflects a ‘no pain, no gain’ and ‘more is better’ approach, where limits are ignored, and sacrifice is seen as the only way to be an athlete. “One step @ a time” instead resonates with values recently identified in a study by Everard and colleagues [62], looking at the injury experiences of track athletes. One of the narratives identified, the ‘longevity narrative’, sees progress from a decrease to an increase in training intensity and wellbeing, which reflects plans and expectations in our participants’ responses. Moreover, the theme “More than sport” could be seen as reflecting some values of narratives such as the ‘discovery narrative’ or ‘more to me narrative’, where participants recognized that sport is not the beginning and end of everything, with other things having priority at times [61,62]. This was particularly important from an ethics of care perspective [63]; when coaches specifically kept their athletes’ mental health post-emergency in higher consideration compared to fitness levels and performance outcomes.

Data collected through a qualitative survey and analyzed using thematic analysis cannot be said to represent cultural narratives. Nevertheless, similarities reflected in the responses of our participants and existing research on injury and athletic identity can illuminate how these findings can be generalized beyond the historically bound experience of the pandemic. Smith [64] discusses the generalizability of qualitative research, and we believe this study could be generalized in two ways. Firstly, through naturalistic generalizability (i.e., when research resonates with the reader’s experience), where sports professionals could recognize their own experience in the themes identified and therefore be able to apply goal-setting strategies in more effective ways. Secondly, these results could be transferred to contexts beyond the pandemic one, for example, to understand goal adjustment following injury or non-normative transitions, in line with the concept of transferability (i.e., a process that occurs when results from one setting are applied to a different one [65]).

Our study has some limitations that future research should consider. First, the voluntary participation in an online self-report evaluation might have excluded those who have lost motivation and disengaged from their goals; moreover, not all participants fully engaged themselves in the process, giving partial or incomplete answers. Second, we did not investigate whether the participants in our study were supported in redefining their goals during this period. Indeed, they could have asked for the support of other team members and staff, because attenuating the social distancing [66] and sharing responsibilities could be beneficial in coping with adversities [67].

Future research should also evaluate the coping strategies used to deal with stressful events that could be associated with setting new goals, examine the effects of the different waves of the COVID-19 pandemic or of the rebooting phase on the goal management of the same population. Finally, it should be considered that those who experience COVID-19 related loss and grief might be susceptible to a range of psychological symptoms, such as potential modifications in their identity and motivation, both in life and in the sports domain, leading them to a lack of or an incorrect definition of their new goals [22]. Derived insights could ultimately facilitate the development of effective psychological approaches to support athletes and coaches in their personal commitment to sport and life in general.

## 5. Conclusions

Notwithstanding the limitations mentioned above, this study reinforced the notion that by making detailed plans, having concrete training programs to follow, and setting performance and process goals to strive for, athletes and coaches could be able to regain more control over their daily structure and athletic life [22]. Moreover, it is vital that coaches consider how their behavior can promote adaptive goals striving, both through a redefinition of a few specific goals (process- and performance-oriented goals), as well as through their everyday engagement and communication with their athletes [15,68]. Indeed, coaches have a key influence in the goal-setting process [45]; however, there is little evidence to support that assigned goals result in greater goal achievement than self-set or participatively-set goals [16]. Therefore, according to the self-concordance model [17], coaches should provide appropriate feedback regarding goal progress, guiding effort and mobilizing resources to the desired goal. In this way, coaches can facilitate optimum goal striving and well-being in their athletes [18].

This study advances our theoretical understanding of the use of goal setting in periods of an outbreak, illustrating how athletes and coaches at different levels reframed their goals. From an applied perspective, it offers an insight into the priorities in reframing process, performance, and outcome goals, highlighting the important role played out by process goals during times of a forced reduced training regime. While this knowledge can be useful in the case of further outbreaks, it can also be transferred to other sports scenarios where athletes (or coaches) are forced to stop or slow down their training (e.g., injury).

## Data Availability

Raw data are available at the following link https://drive.google.com/drive/folders/15_PE_B2RKauHmSgY9XVajkfJlYDtZJ1e?usp=sharing. The scales used for the mixed-method project will be made available by the first author upon reasonable request.
